# The lipidome of the kidney tubules of ob/ob mice is affected at the infralesional level

**DOI:** 10.1038/s41420-025-02827-9

**Published:** 2025-11-10

**Authors:** Lydie Cheval, Julio L. Sampaio, Virginie Poindessous, Gilles Crambert, Nicolas Pallet

**Affiliations:** 1 Laboratoire de Physiologie Rénale et Tubulopathies, Centre de Recherche des Cordeliers, INSERM, Sorbonne Université, Université Paris Cité, Paris, France; 2 CNRS EMR 8228 - Unité Métabolisme et Physiologie Rénale, Paris, France; 3 CurieCoreTech Metabolomics and Lipidomics Technology Platform, Institut Curie, Paris, France; 4 Centre de Recherche des Cordeliers, INSERM U1138, Université Paris Cité, Paris, France; 5 Department of Clinical Chemistry, Assistance Publique Hôpitaux de Paris, Georges Pompidou European Hospital, Paris, France

**Keywords:** Kidney, Lipidomics

## Abstract

Whether changes in tubular lipidomic composition precede the onset of observable kidney damage in obesity is unknown. We manually isolated the proximal convoluted tubule (PCT), the cortical thick ascending limb of Henle’s loop (cTAL) and the cortical collecting duct (CCD) from five lean and five ob/ob mutant mice that have a defect in the leptin gene and subjected them to lipidomic analysis by high-resolution mass spectrometry acquisition. In total, more than 700 molecular species of glycerophospholipids (including cardiolipins and peroxidised phospholipids) and sphingolipids were identified and analyzed. Significant compositional differences were observed between the three tubular segments, which serve as true signatures associated with obesity. The fatty acid composition of triglycerides, phospholipids and cardiolipins was significantly altered by leptin deficiency and these changes were predominantly observed in the polyunsaturated fatty acid (PUFA) composition and showed different patterns in different tubular segments. This resource provides unparalleled details of the lipid composition of tubular segments, thereby enhancing our understanding of the tubular metabolic and lipidomic reprogramming that occurs at the earliest stages of nephropathy associated with obesity in ob/ob mice.

## Introduction

An analysis of the pathophysiological processes occurring in the initial stages of kidney disease enables to understand the close relationship between the causal systemic disturbances associated with obesity and the metabolic/lipidic reprogramming that takes place in the tubules in the subclinical and sublesional phases. This excludes the role of other signaling pathways associated with inflammation, fibrosis or cell death, which may also have an impact on metabolic and lipid homeostasis in the tubules.

The tubular system within the kidney is constituted of discrete segments that exhibit distinctive cellular and functional phenotypes, thereby supporting exclusive physiological functions that are reflected in distinct transcriptomic, proteomic and lipidomic identities [1–3]. Obesity is a medical condition that result in multisystem disturbances, leading to chronic kidney disease (CKD). These conditions exert a significant influence on energy metabolism and lipidomic composition, predominantly affecting the phenotype and functions of the tubule segments [4–9]. From the perspective of lipidomics applied to isolated tubules, the utilization of cutting-edge lipidomic analysis platforms, coupled with microdissection or isolation techniques, offers a unique opportunity to undertake comparative studies of tubular lipid composition across a range of conditions. In particular, lipidome analysis techniques need to be adapted to the experimental conditions, especially the very small amounts of material available for analysis in this setting.

Previously, and for the first time, we carried out a complete lipidomic analysis by tandem mass spectrometry of three isolated tubular segments: proximal convoluted tubule (PCT), cortical ascending thick limb of Henle (CTAL), and cortical collecting duct (CCD) [3]. This study led to the identification of different compositions among the three tubular segments, which may be considered as true signatures. The data also showed that the lipid composition at the class level of these tubular segments is affected in obese mice [3].

The objective of this study is to provide a comprehensive characterization of the lipidomic composition of the PCT, CTAL and CCD segments in ob/ob mutant mice at the infralesional stage. The genetic leptin-deficient ob/ob mice and are widely used as animal models to study obesity and related metabolic disorders. This included oxo-phospholipidomics and a thorough examination of glycerophospholipids including cardiolipins, and sphingolipids. Our findings are key to understanding the initial biochemical alterations that occur in kidney tubules at the early stages of obesity associated nephropathy in ob/ob mice with leptin deficiency.

## Results

### B6.V-Lep ob/ob JRj mice are obese, diabetic, with a normal renal function and no histological damage

**Fig. 1:**
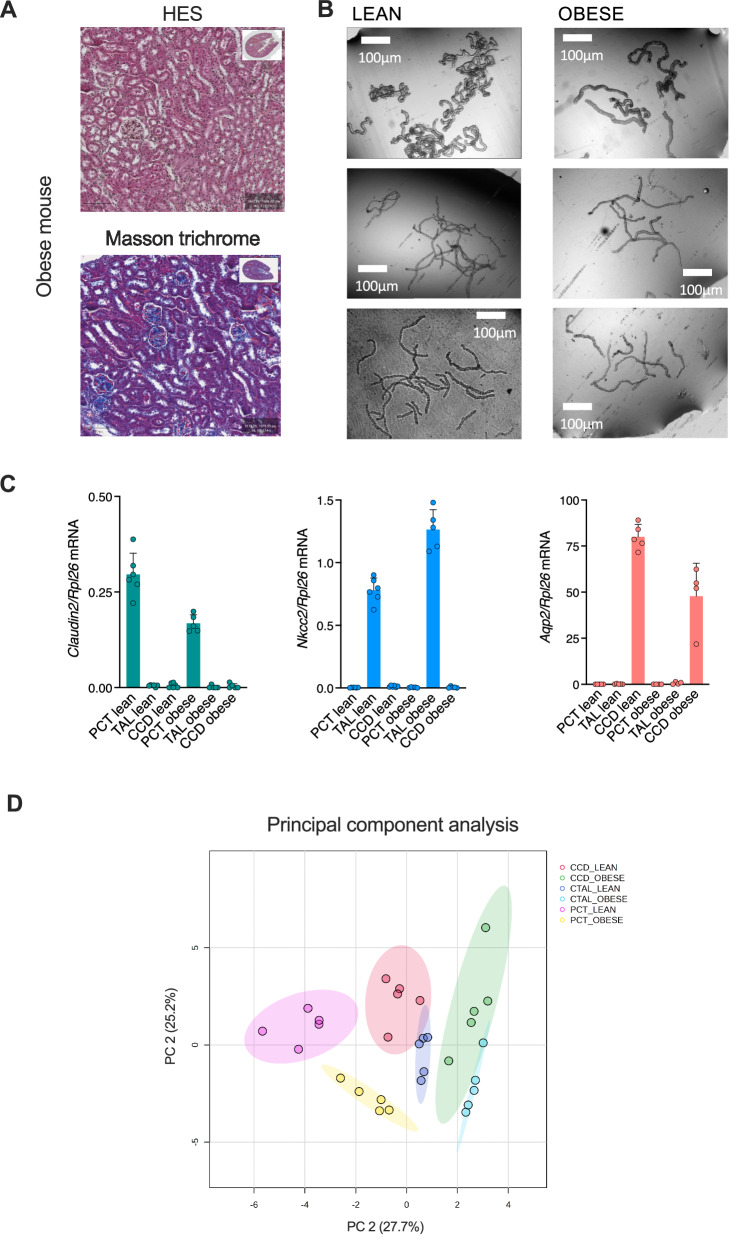
B6.V-Lep ob/ob JRj are obese, diabetic, with a normal renal function and no histological damage. **A** Representative photomicrograph of a kidney section from an ob/ob mouse after staining with haematoxilin-eosin-safran (left) and after staining with Masson’s trichrome (right). The cortical tubules of the contralateral kidney were dissected for lipidomic analysis. Original magnification of ×10. **B** Phase contrast micrographs of isolated tubule segments from lean and obese mice at original magnification of ×40. **C** Relative expression levels of *Cldn2*, *Slc12a1*, and *Aqp2* as markers of tubule segment origin using RT-qPCR. Rpl26 was used as a housekeeping gene and five samples were taken per segment. **D** Dimension reduction by principal component analysis on all identified lipid classes in the three segments from five obese and five lean mice. The resulting plot shows the 2D scores between the selected principal components that best explain the lipid variance.

Five leptin-deficient obese (B6.V-Lep ob/ob JRj) male mice were used in this study. These mice were 12 weeks old. They weighed on average 46.3 ± 1.8 g (mean baseline value for normal 4 months C57BL/6J males ~35 g [10], and the glycemia was 21.6 ± 7.5 mmol/L (mean baseline value for normal 4–8 months C57BL/6J males ~7.4 mmol/L [10]); their renal function was defined by a plasma creatinine concentration of 6.6 ± 0.9 μmol/L (mean baseline value for normal 4–8 months C57BL/6J males ~10.2 μmol/L [10]), a blood urea nitrogen concentration of 8.1 ± 1.3 mmol/L (mean baseline value for normal 4–8 months C57BL/6J males ~9 mmol/L [10]), and an average proteinuria of 0.9 ± 0.6 g/L (mean baseline value for normal 4–8 months C57BL/6J males ~0.027 g/L). An examination of the kidney cortex following the application of haematoxylin eosin staining (which provides information regarding the pattern, shape, and structure of cells) and Masson trichrome (which stains in blue collagen fibers) did not evidence any structural anomalies (Fig. 1A). Thus, these obese mice were overtly diabetic, yet demonstrated normal renal function, moderate proteinuria (which may indicate glomerular hyperfiltration, as seen in obesity and in the early stages of diabetic nephropathy), and no histologically visible tubular or glomerular lesion.

Three tubular segments (PCT, CTAL and CCD) were isolated from obese and lean mice using the liberase and protease perfusion and digestion protocol, which has been successfully applied for lipidomic profiling [3, 11]. The phase-contrast microscopy examination corroborated the origin of the three segments based on their morphologic characteristics. The PCT was observed to be large and convoluted, the CTAL segments were noted to be thin, bright, and straight, while the CCD appeared dense with a cobblestone appearance (Fig. 1B). Furthermore, these three segments exhibited specific differentiation markers. The expression of *Cldn2* (encoding Claudin-2) was observed in the PCT, whereas *Slc12a1* (encoding Nkcc2) and *Aqp2* (encoding Aquaporin-2) were not expressed. *Slc12a1* was expressed in CTAL, whereas *Cldn2* and *Aqp2* were not. In contrast, Aqp2 was expressed in CCD, while *Cldn2* and *Slc12a1* were not, as illustrated in Fig. 1C.

The contents of the lipids class and species were normalized to the total identified lipids and expressed as a proportion of the whole lipid content in a sample. A principal component analysis of all the lipid molecular classes identified in the samples revealed that each of them segregated into three distinct groups according to their segments of origin, and distinguished obese from their leaner counterparts (Fig. 1D). Furthermore, the replicates demonstrated high consistency. These results indicate that each of the 3 ob/ob mouse tubular segments analyzed had a distinct lipidomic signature within each other and with lean tubular segments.

### Leptin deficiency gives each tubular segment a lipid signature

**Fig. 2:**
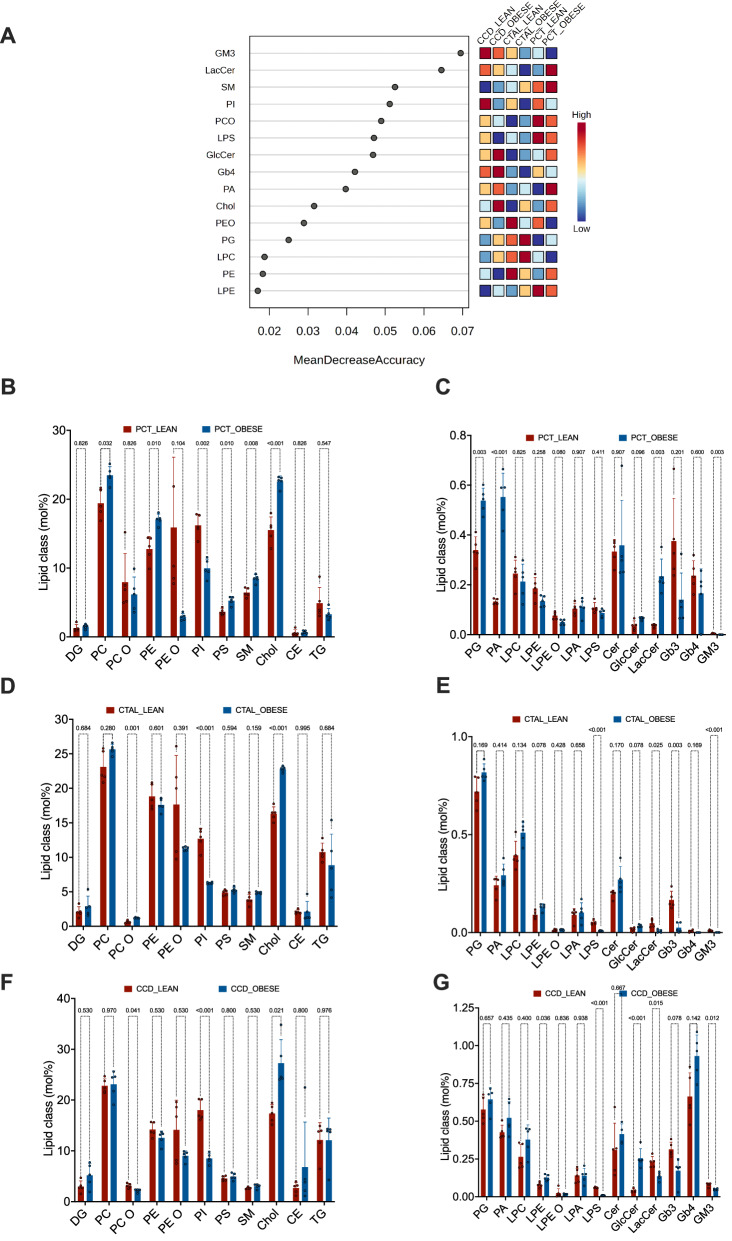
Leptin deficiency gives each tubular segment a lipid signature. **A** Significant lipid classes in the tubule segments identified by Random Forest classification between lean and obese mice. Lipid class importance (top 15) is calculated by Mean Decrease Accuracy for classification between lean group and ob/ob group. ntree = 500, OOB error rate = 0 and class.error = 0. The features ((a lipid class) are ranked by the mean decrease in classification accuracy after permutation. Dark red indicates that a feature (a lipid class) is enriched in a group. **B** Histograms of the proportional distribution of major lipid classes in the composition of PCT, isolated from 5 lean and 5 ob/ob mouse kidneys for classes >1 mol%. The percentage of total membrane lipids in a sample, expressed in mol%, is shown on the y-axis. *p* values were computed with multiple *T* tests corrected with the Holm-Šídák method. **C** Histograms of the proportional distribution of major lipid classes in the composition of PCT, isolated from 5 lean and 5 ob/ob mouse kidneys for classes <1 mol%. The percentage of total membrane lipids in a sample, expressed in mol%, is shown on the y-axis. *p* values were computed with multiple *T* tests corrected with the Holm-Šídák method. **D** Histograms of the proportional distribution of major lipid classes in the composition of CTAL, isolated from 5 lean and 5 ob/ob mouse kidneys for classes >1 mol%. The percentage of total membrane lipids in a sample, expressed in mol%, is shown on the y-axis. *p* values were computed with multiple *T* tests corrected with the Holm-Šídák method. **E** Histograms of the proportional distribution of major lipid classes in the composition of CTAL, isolated from 5 lean and 5 ob/ob mouse kidneys for classes <1 mol%. The percentage of total membrane lipids in a sample, expressed in mol%, is shown on the y-axis. *p* values were computed with multiple *T* tests corrected with the Holm-Šídák method. **F** Histograms of the proportional distribution of major lipid classes in the composition of CCD, isolated from 5 lean and 5 obese mouse kidneys for classes >1 mol%. The percentage of total membrane lipids in a sample, expressed in mol%, is shown on the y-axis. *p* values were computed with multiple *T* tests corrected with the Holm-Šídák method. **G** Histograms of the proportional distribution of major lipid classes in the composition of CCD, isolated from 5 lean and 5 obese mouse kidneys for classes <1 mol%. The percentage of total membrane lipids in a sample, expressed in mol%, is shown on the y-axis. *p* values were computed with multiple *T* tests corrected with the Holm-Šídák method.

We then conducted a comparative analysis of the lipid class composition of the three segments between 5 lean and 5 leptin mutant mice. Lean mice were 12 weeks old males of the C57BL/6jRj genetic background. Our initial objective was to ascertain whether obesity imprinted a distinctive signature on each tubular segment. To achieve this, the distribution of lipid classes was subjected to analysis. A Random Forest classifier was employed to identify the most significant lipid classes predictive of the three tubule segment groups, with and without obesity (Fig. 2A). This approach makes it possible to assign a lipid signature to each tubular segment analyzed and to see whether obesity has an effect on this signature. For example, a high level of lactosylceramide (LacCer) and sphingomyelin (SM) discriminates obese PCT; a high level of phosphatidylglycerol (PG) and lysophosphatidylcholine (LPC) is a characteristic feature of obese CTAL, whereas high levels of glucosylceramide (GlcCer) and Gb4 globosides are characteristic of obese CCD.

Quantitative comparative analysis of lipid class levels provided additional information on the specific impact of obesity on each segment. Obesity significantly affected the cellular contents of the vast majority of the classes analyzed (Fig. 2B–G). Moreover, the amplitude and direction of variation between the two conditions appeared to be specific to a class of lipids and the segments analyzed. For instance, the PC and PA contents were markedly elevated in PCT of obese relative to lean subjects, whereas no discernible variation was evident in CTAL and CCD. In contrast, the levels of GlcCer were specifically reduced in CCD of obese mice, but not in CTAL and PCT. Furthermore, certain classes, such as non-esterified cholesterol or the ganglioside GM3 demonstrated an increase and a decrease, respectively, in all three obese tubule segments.

Thus, our comparative lipidomic analysis suggests that obesity profoundly impacts the lipid composition of the PCT, CTAL and CCD, in amplitudes and directions that are dependent on the tubular segments under investigation. These signatures indicate intrinsic differences in biosynthetic and metabolic activities.

### Leptin deficiency affects triglyceride composition in fatty acids length and saturation

Subsequently, we conducted detailed analyses of the distribution of specific lipid molecular species in accordance with conditions and tubular segments, thereby acquiring a more functional perspective. Triacylglyceride (TG) is a neutral lipid that accumulates in cells as fatty acids (FA) reserves in anhydrous lipid droplets. FA can be released from TG in order to produce metabolic energy or membrane building blocks. Lipidomic studies indicate that neutral lipids such as TG accumulate in diabetic kidneys [12, 13]. We then studied the quantitative and qualitative aspects of composition of TG according to the length of the FA chains and their degree of saturation. The majority of fatty acid species (with 16, 18, 20 and 22 carbons) have been the focus of our analyses, as certain molecular species identified by the software but representing a minority (e.g. those with an odd number of carbons) may be false positives due to the high sensitivity of the technique.

**Fig. 3:**
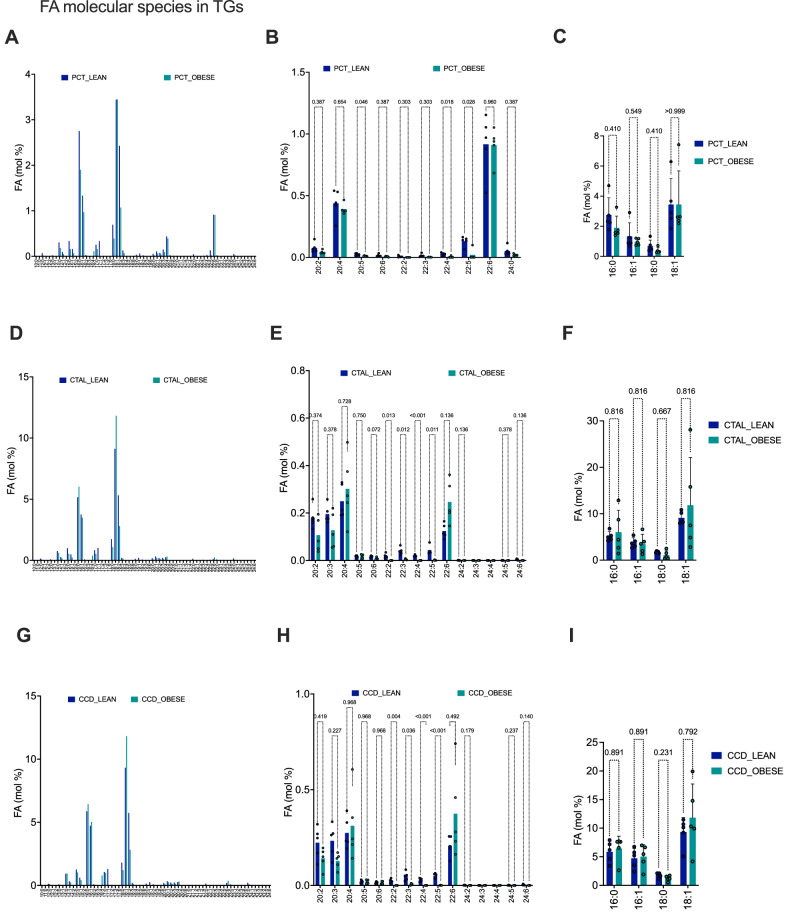
Leptin deficiency affects triglyceride composition in fatty acids length and saturation. **A** Histograms of the proportional distribution of all the fatty acids identified in the composition of TG in PCT isolated from 5 lean and 5 ob/ob mouse kidneys. The percentage of total membrane lipids in a sample, expressed in mol%, is shown on the y-axis. **B** Histograms of the proportional distribution of the 20, 22 and 24 carbons fatty acids identified in the composition of TG in PCT isolated from 5 lean and 5 ob/ob mouse kidneys. The percentage of total membrane lipids in a sample, expressed in mol%, is shown on the y-axis. *p* values were computed with multiple *T* tests corrected with the Holm-Šídák method. **C** Histograms of the proportional distribution of the 16 and 18 carbons saturated and monounsaturated fatty acids identified in the composition of TG in PCT isolated from 5 lean and 5 ob/ob mouse kidneys. The percentage of total membrane lipids in a sample, expressed in mol%, is shown on the y-axis. *p* values were computed with multiple T tests corrected with the Holm-Šídák method. **D** Histograms of the proportional distribution of all the fatty acids identified in the composition of TG in CTAL isolated from 5 lean and 5 ob/ob mouse kidneys. The percentage of total membrane lipids in a sample, expressed in mol%, is shown on the y-axis. **E** Histograms of the proportional distribution of the 20, 22 and 24 carbons fatty acids identified in the composition of TG in CTAL isolated from 5 lean and 5 ob/ob mouse kidneys. The percentage of total membrane lipids in a sample, expressed in mol%, is shown on the y-axis. *p* values were computed with multiple *T* tests corrected with the Holm-Šídák method. **F** Histograms of the proportional distribution of the 16 and 18 carbons saturated and monounsaturated fatty acids identified in the composition of TG in CTAL isolated from 5 lean and 5 ob/ob mouse kidneys. The percentage of total membrane lipids in a sample, expressed in mol%, is shown on the y-axis. *p* values were computed with multiple *T* tests corrected with the Holm-Šídák method. **G** Histograms of the proportional distribution of all the fatty acids identified in the composition of TG in CCD isolated from 5 lean and 5 ob/ob mouse kidneys. The percentage of total membrane lipids in a sample, expressed in mol%, is shown on the y-axis. **H** Histograms of the proportional distribution of the 20, 22 and 24 carbons fatty acids identified in the composition of TG in CCD isolated from 5 lean and 5 ob/ob mouse kidneys. The percentage of total membrane lipids in a sample, expressed in mol%, is shown on the y-axis. *p* values were computed with multiple *T* tests corrected with the Holm-Šídák method. **I** Histograms of the proportional distribution of the 16 and 18 carbons saturated and monounsaturated fatty acids identified in the composition of TG in CCD isolated from 5 lean and 5 ob/ob mouse kidneys. The percentage of total membrane lipids in a sample, expressed in mol%, is shown on the y-axis. *p* values were computed with multiple *T* tests corrected with the Holm-Šídák method.

A global analysis of the distribution of FA species contributing to TG molecules revealed an increase in the quantity of TG contents in distal tubular segments compared to PCT, but obesity did not exert any influence on the TG contents of tubule segments (Fig. 3A, D, G). Moreover, PCT exhibited a higher enrichment of long-chain FA with 20 and 22 carbons compared to CTAL and CCD (Fig. 3A, D, G).

Variations in the constitution of TG in the long-chain polyunsaturated FA (C20, C22 and C22 PUFA) were discerned between the lean and ob/ob mice, with respect to the tubule segments. Notably, PUFA might protect against the development of kidney disease [13]. The CTAL and CCD of obese mice demonstrated in the great majority of the cases a reduction in the number of PUFA present in their TGs in comparison with lean mice, while the PCT of lean mice exhibited a more limited alteration in the distribution of PUFA in response to obesity (Fig. 3B, E H). The FA 22:4 (adrenic acid, a source of prostaglandins) was virtually absent in TGs of the tubule segments of ob/ob mice (Fig. 3B, E, H). The FA 22:6 (docosahexanoic acid, which is thought to have anti-inflammatory and anti-fibrotic properties [14]) was enriched in TGs of CTAL but not in PCT and CCD of ob/ob mice compared to lean mice (Fig. 3B, E, H). The FA 20:5 (eicosapentaenoic acid, which may ameliorates diabetic nephropathy in mouse [15]) was significantly depleted in TGs of PCT of ob/ob mice compared to lean mice (Fig. 3B, E, H). Finally, the tubule contents of TG in FA 20:4 (arachidonic acid) were not affected by obesity.

The composition of saturated FA, such as palmitate (C16:0), was analyzed (Fig. 3C, F, I). High concentrations of palmitate have been linked to a number of adverse conditions, as the release of palmitate during the mobilization of stored fat can have a detrimental impact on cellular function [16]. Saturated FA contents were not specifically enriched in TGs in the tubular segments of ob/ob mice compared to lean mice, nor was the case for more beneficial monounsaturated FA, such as oleic acid (C18:1), which was not found to be differentially distributed in TG of tubular segments from ob/ob mice compared to lean mice.

These findings demonstrate that at the earliest stages of kidney disease, leptin deficiency does not impact the overall TG quantity in tubules, but does influence the composition of PUFA. In this model, the proportion of saturated and monounsaturated FA remains unaltered.

### The phospholipidomic profile of tubules from ob/ob mice is remodeled without sensitization to ferroptosis

**Fig. 4:**
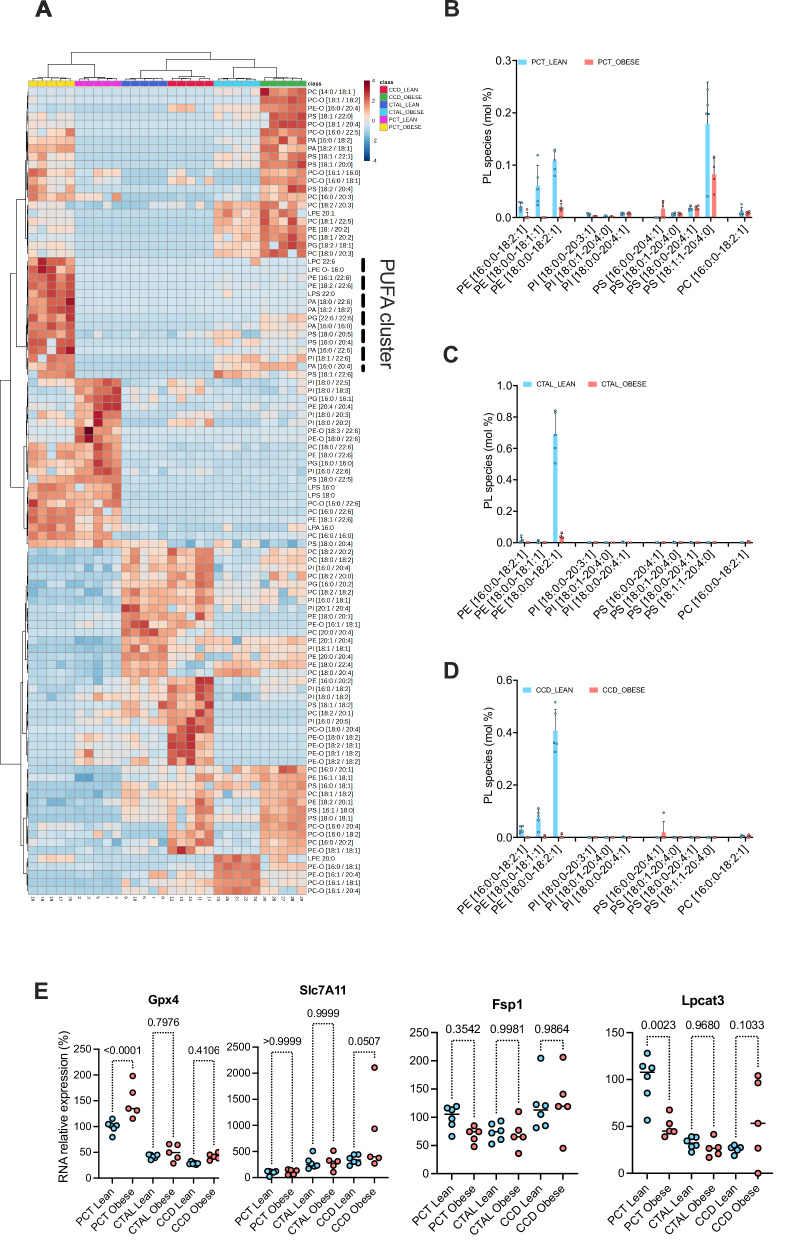
The phospholipidomic profile of tubules from ob/ob mice is remodeled without sensitization to ferroptosis. **A** Hierarchical clustering of the 100 most differentially expressed lipid molecular species among the three tubular segments (one-way ANOVA with five replicates). Each row represents the relative lipid molecular species composition, while each column represents either a replicate or a condition. Samples were normalized to sum and each metabolite was autoscaled (mean-centered and divided by standard deviation of each variable). The cluster enriched in long and very long chain PUFA and specific for PCT obese is shown. **B** Histograms of the distribution of different peroxidized phospholipids lipid classes including oxo-phosphatidylethanolamine (PE), oxo-phosphatidylinositol (PI), oxo-phosphatidylserine (PS) and oxo-phosphatidylcholine (PC), in the PCT of 5 lean and 5 ob/ob mouse kidneys. The y-axis indicates the relative expression of each lipid class (mol%), which is the percentage of total membrane lipids in the sample. **C** Histograms of the distribution of different peroxidized phospholipids lipid classes including oxo-phosphatidylethanolamine (PE), oxo-phosphatidylinositol (PI), oxo-phosphatidylserine (PS) and oxo-phosphatidylcholine (PC) in the CTAL of 5 lean and 5 ob/ob mouse kidneys. The y-axis indicates the relative expression of each lipid class (mol%), which is the percentage of total membrane lipids in the sample. **D** Histograms of the distribution of different peroxidized phospholipids lipid classes including oxo-phosphatidylethanolamine (PE), oxo-phosphatidylinositol (PI), oxo-phosphatidylserine (PS) and oxo-phosphatidylcholine (PC) in the CCD of 5 lean and 5 ob/ob mouse kidneys. The y-axis indicates the relative expression of each lipid class (mol%), which is the percentage of total membrane lipids in the sample. **E** *Gpx4, Slc7a11, Fsp1 and Lpcat3* transcripts measured by RT-qPCR of mRNA from PCT, CTAL and CCD isolated from 5 lean and 5 ob/ob mice. *p* values were computed with a one-way ANOVA followed by a Šídák’s multiple comparisons test.

We next conducted a comparative analysis of the phospholipidome (phospholipids, ether phospholipids and lysophospholipids in particular) to ascertain whether leptin deficiency produces a distinctive tubular signature. This information is significant given that PUFA membrane composition determines sensitivity to lipid peroxidation during a process of programmed cell death known as ferroptosis [17]. Ferroptosis has been demonstrated to be involved in the development and progression of diabetic nephropathy [18]. We performed a hierarchical clustering of the 100 molecular species with the highest distribution variance among the conditions. The results demonstrated that the PCT exhibited a distinct clustering pattern from the more distal segments. Of particular note was the observation that the distal segments of ob/ob mice (CTAL and CCD) formed a cluster on one side, while the lean segments were clustered on the other. The findings indicate that leptin deficiency exerts an influence on the tubular phospholipidome, resulting in a loss of the specific lipidomic identity of CTAL and CCD (Fig. 4A).

A highly specific pattern was observed in ob/ob mice PCT comprising a cluster of long and very long chain PUFA: PE [16:1/22:6], PE [18:2/22:6], PA [18:0/22:6], PA [18:2/18:2], PG [22:6/22:6], PA [16:0/16:0], PS [18:0/20:5], PS [16:0/20:4], PA [16:0/22:6] and PI [18:1/22:6] (Fig. 4A). However, the species most susceptible to peroxidation are PE, PI and PC, which are not overrepresented in this group, nor are FA 20:4 and 22:4, which are the prime FA targeted by ferroptosis [19, 20].

In order to identify the proferroptotic oxygenated phospholipids, a redox phospholipidomics analysis was performed. The analysis revealed the presence of a number of oxygenated PC, PS, PI and PE, albeit in smaller quantities in the tubular segments of ob/ob mice compared to lean mice (Fig. 4B, C, D).

To substantiate these observations, we performed an analysis of the relative expression of the gene transcripts of three master regulators of ferroptosis in cells: glutathione peroxidase 4 (Gpx4), solute carrier (Slc) 7A11) and ferroptosis suppressor protein 1 (Fsp1) (Fig. 4E). Gpx4 is a phospholipid hydroperoxidase that plays a critical role in protecting cells from membrane lipid peroxidation. The cystine/glutamate antiporter Slc7A11 is responsible for importing cystine, which is used for glutathione synthesis and maintenance of antioxidant defenses. Fsp1 is a glutathione-independent ferroptosis inhibitor molecule. Gpx4 was significantly induced in ob/ob PCT, whereas Slc7A11 was more modestly expressed in ob/ob CCD, suggesting that antiferroptotic systems are engaged in obese tubules. Finally, we analyzed the expression of lysophosphatidylcholine acyltransferase 3 (Lpcat3), a key enzyme in the induction of ferroptosis with a putative role in regulating the metabolism of arachidonic acid (FA 20:4) to produce 20:4-phospholipids, and found that its expression was significantly reduced in ob/ob PCT (Fig. 4E).

Together, these findings indicate that the phospholipid compositional profile of the tubular segments of ob/ob mice, and notably the PCT, appear to exhibit resilience to lipid peroxidation. This resilience may be attributed to favorable transcriptional reprogramming.

### Cardiolipin composition is profoundly remodeled in ob/ob mice

Cardiolipin (CL) is a distinctive phospholipid that is localized and synthesized in the inner mitochondrial membrane. CL plays a pivotal role in mitochondrial metabolism, ensuring the optimal structural and morphological integrity of mitochondrial membranes and regulating the activity of a multitude of proteins and enzymes that are crucial for mitochondrial function. Furthermore, the length and degree of saturation of CL acyl chains appear to affect mitochondrial fusion, with longer chains and higher unsaturation levels promoting this process [21]. Given the critical role of dysfunctional mitochondria in diabetic kidney disease [22], we performed a comparative analysis of CL chain length and saturation between tubule segments of lean and ob/ob mice.

**Fig. 5:**
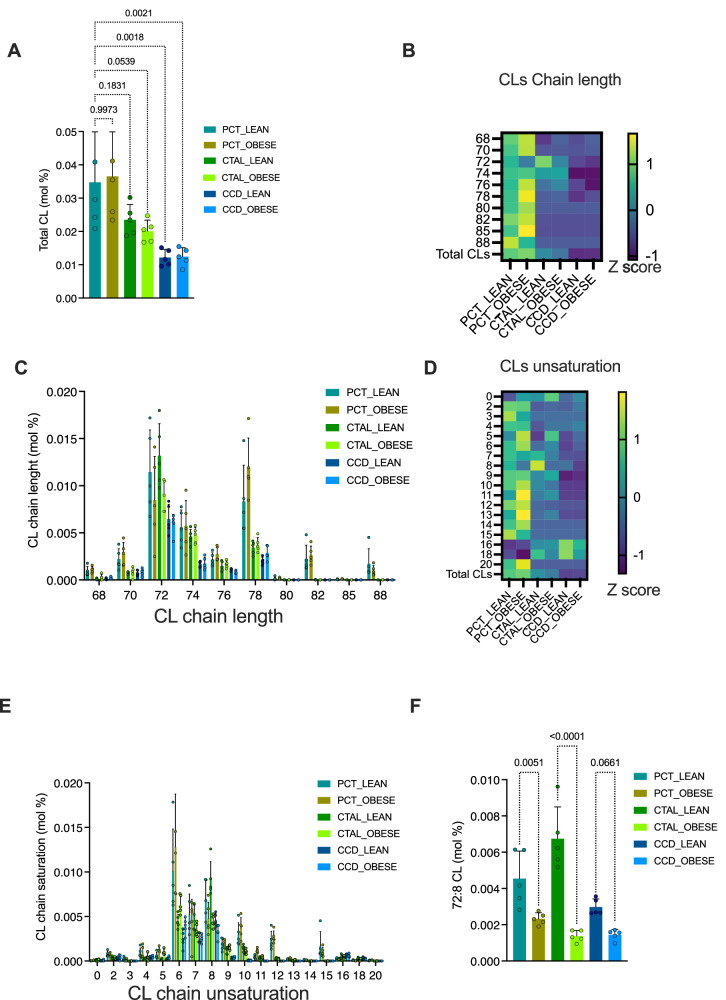
Cardiolipin composition is profoundly remodeled in ob/ob mice. **A** Histograms of the distribution of cardiolipin (CL) contents of PCT, CTAL and CCD of 5 lean and 5 ob/ob mouse kidneys. The y-axis indicates the relative expression of each lipid class (mol%), which is the percentage of total membrane lipids in the sample. *p* values were computed with a one-way ANOVA followed by a Dunnett’s multiple comparisons test. **B** Heat map showing the distribution of the sum of chain lengths of fatty acids composing cardioliplins (CL) in the three tubule segments (with *n* = 5 replicates per segment) of lean and ob/ob mice. Each feature is a normalized data (Z-score): each variable is mean centered and divided by the standard deviation. **C** Histograms of the distribution of the sum of chain lengths of fatty acids composing cardioliplins (CL) in the three tubule segments (with *n* = 5 replicates per segment) of lean and ob/ob mice. The y-axis indicates the relative expression of each lipid class (mol%), which is the percentage of total membrane lipids in the sample. **D** Heat map showing the distribution of the sum of hydrocarbons unsaturations of fatty acids composing cardioliplins (CL) in the three tubule segments (with *n* = 5 replicates per segment) of lean and ob/ob mice. Each feature is a normalized data (Z-score): each variable is mean centered and divided by the standard deviation. **E** Histograms of the distribution of the sum of hydrocarbons unsaturations of fatty acids composing cardioliplins (CL) in the three tubule segments (with *n* = 5 replicates per segment) of lean and ob/ob mice. The y-axis indicates the relative expression of each lipid class (mol%), which is the percentage of total membrane lipids in the sample. **F** Histograms of the distribution of tetra-linoleoyl cardiolipins (72:8 CL) contents of PCT, CTAL and CCD of 5 lean and 5 ob/ob mouse kidneys. The y-axis indicates the relative expression of each lipid class (mol%), which is the percentage of total membrane lipids in the sample. *p* values were computed with a one-way ANOVA followed by a Šídák multiple comparisons test.

Firstly, PCT appeared to be enriched in CL overall in comparison with the other segments, which may be indicative of an increase in the cellular contents of mitochondria in this segment [23] (Fig. 5A, B). Leptin deficiency did not impact the global contents in CL within each tubule segments. Secondly, ob/ob PCT exhibited an enrichment in long-chain CL (eg. accounting for >76 carbons) with a high degree of unsaturation (>10 unsaturations), a phenomenon that is not observed in the CTAL and CCD segments of ob/ob mice. It should be noted, however, that CLs with these long PUFA are not quantitively the most representative of CL in tubule segments, compared with CL with 72. Noteworthy, CTAL and CCD were found to be enriched with CL at 18 and 20 unsaturations, in comparison to PCT. The biological significance of this observation remains to be demonstrated (Fig. 5C, D).

Finally, we examined the distribution of 72:8 CL (tetralinoleoyl-CL), which are theoretically composed of four linoleic FA (FA 18:2) (Fig. 5E). These specific CL are critical for the optimal functioning of mitochondrial transporters, including the ADP/ATP transporter (ANT), which plays an important role in the transfer of ATP generated by oxidative phosphorylation from the inner mitochondrial membrane to the intermembrane space [24]. The proportion of 72:8 CL was found to be significantly reduced in the three tubular segments examined in ob/ob mice compared with lean mice (Fig. 5F).

These findings collectively indicate that leptin deficiency exerts a profound impact on the composition of CL. The CL of PCT in ob/ob mice display a distinctive compositional profile, characterized by the presence of long-chain, highly unsaturated FA. Furthermore, leptin deficiency was associated with a reduction in 72:8 CL in all tubule segments, which may reflect a mitochondrial dysfunction.

## Discussion

This study presents the first comprehensive analysis of the compositional changes in the lipidome of the PCT, CTAL and CCD of ob/ob mice. Our results demonstrate that qualitative and quantitative alterations occur at an early stage in the development of nephropathy, at infrahistological and sub-clinical levels. Given the cellular functions of the molecular species identified, it is highly probable that they are involved in kidney pathophysiological processes, although the nature and mechanisms of these processes remain to be elucidated.

A noteworthy discovery of the study is the identification of distinct compositions among the three tubular segments of ob/ob mice, and which distinguishes them from lean mouse tubules, which can be regarded as signatures of a diseased state. These intrinsic lipidomic characteristics, potentially supported by a specific metabolic reprogramming and likely involved in the initial stages of kidney disease, are consistent with the hypothesis of a lipotype [25]. Modifications in lipid metabolism have the potential to alter cell type composition and induce abnormalities in tissue function and structure [9, 26–29]. Consequently, the integration of multi-omics data (transcriptomic, proteomics and lipidomics) represents a promising avenue for conducting comprehensive studies to determine the causes and mechanisms of this lipidomic reprogramming, as well as its functional consequences.

This is a crucial consideration in the context of our findings. For example, we found that the tubular composition in TG of ob/ob mice did not quantitatively differ from lean mice, whereas it has been previously established that obesity associated with an accumulation of TG in diseased tissues [13, 30, 31]. However, our findings revealed more subtle alterations in FA composition, specifically a notable reduction in PUFA in TG from ob/ob segments, particularly in the distal segments, CTAL and CCD. The simplest explanation for this observation is that TG cycling is the process of continuous degradation and re-synthesis of TG in cellular stores [32, 33]. This process facilitates the conversion of saturated FA to more saturated FA through a series of cycles and modifications. The overall result is an adjustment of the stored FA pool to meet the changing needs of the cell. The question of how this process differentially operates between ob/ob and lean tubules remains to be tested in dedicated studies. However, it is a possibility that this will occur before the deregulation of FA metabolism results in the accumulation of TGs.

Furthermore, our data offer new insights into tubular phospholipid remodeling in the kidney tubule during obesity associated with leptin deficiency. We conducted a detailed examination of the composition of long-chain PUFA-PLs, which are targets of lipid peroxidation during ferroptosis [34]. This regulated cell death process has been identified as a significant contributor to the progression of a number of kidney diseases, particularly diabetes-associated nephropathy [18]. Our findings indicate that, despite a clear link between leptin deficiency and significant alterations in the phospholipid composition of the tubular segments examined, there is no discernible indication of an elevated susceptibility to lipid peroxidation, particularly at the initial stages of renal disease, when the analysis was conducted. The very low presence of peroxidised phospholipids in the tubular segments of ob/ob mice compared to lean mice supports this conclusion. It must be acknowledged that the possibility of ferroptosis and PUFA peroxidation playing a role in the pathophysiology of diabetic/obese kidney disease at a later stage cannot be discounted. However, our data suggest that these processes may be effectively contained at very early stages by the implementation of an effective cytoprotective enzyme regulation program.

Our study represents the first characterization of compositional remodeling of CL in tubular segments of ob/ob mice, which revealed an increased in PUFA-containing CL in PCT and a reduction in the contents in 72:8 CL, which presumably correspond to tetralinoleoyl CL (4× FA18:2). It is of critical importance to note that diabetes is associated with CL remodeling, which results in the replacement of linoleic FA (18:2) with PUFA in CL, which is corroborated by our results [35, 36]. Critically, PUFA in CL are prone to lipid peroxidation due to the high number of unsaturated carbons, and are associated with mitochondrial dysfunction and apoptosis [36]. In the other hand, CL interact with several mitochondrial carrier proteins, including the ANT, the pyruvate carrier, the tricarboxylate carrier and the carnitine/acylcarnitine translocase [37]. The activity of the ANT has been demonstrated to be optimal only in the presence of tetralinoleoyl-CL, whereas other CL species were ineffective in catalyzing ANT activity [24]. Finally, the length and degree of saturation of CL acyl chains appear to impact mitochondrial fusion, with longer chains and higher unsaturation levels facilitating this process [21]. Although a matter of contention, fused mitochondria confer cytoprotection and allows the distribution of matrix components and the stimulation of oxidative phosphorylation activity. It remains to be established whether the changes in CL composition observed in the tubule segments of obese mice are responsible for mitochondrial and tubular dysfunction, or whether they merely reflect mitochondrial fitness states.

Limitations of our work include the lack of kinetic analysis of tubular lipidomic reprogramming over time and progression to chronic lesions with histological and clinical correlation. Furthermore, the data are purely descriptive and the mechanistic hypotheses remain to be demonstrated, but this research paves the way for a multitude of avenues of investigation in the field of tubular lipid metabolism and kidney disease. Moreover, leptin can regulate lipid metabolism, our model does not allow us to specifically define what aspects of tubular lipidome changes are related to obesity and leptin deficiency. Finally, the use of male mice is also a limitation, as it does not allow for the impact of oestrogens on the regulation of lipid metabolism in the kidney to be taken into account.

Shotgun lipidomics, like any other technique, has its advantages and disadvantages, and thorough validation is required to know the precise experimental conditions under which it should be used. Shotgun lipidomics is highly sensitive, allowing the analysis of small amounts of sample; it is highly specific, especially when combined with tandem MS and high mass resolution; it allows quantitative analysis through the inclusion of internal standards co-incubated with endogenous lipids, as opposed to LCMS; and it allows rapid sample preparation and acquisition, allowing high throughput. The disadvantages of this technique are related to ion suppression, but this is overcome by the fact that different lipid classes have different ionisation properties, which can be exploited by adding different infusion solvents and exploring both polarity modes [38]. For isobaric species, most isobaric species can now be resolved using Orbitrap technology with resolutions greater than 200k [39]. Finally, artefacts related to in-source fragmentation can be avoided by infusing synthetic standards where the source conditions have been optimized to avoid this phenomenon [40]. The main reason for using this approach in our study was the small amount of sample available. Kidney tubules are very small structures and for a complete lipidomics analysis using this approach, we collected a few hundred mm of tubule replicate sample. The amount that would need to be collected for an LCMS experiment would make this experiment highly imprecise. For the complete analysis, we performed 3 independent and complementary acquisitions in both polarities per sample, allowing us to study the differential ionisation of the different lipid classes. The inclusion of internal standards allowed us to combine all acquisitions into a comprehensive and quantitative lipid profile covering the main lipid classes in the samples. The combination of high mass resolution (280k) and the known fragmentation behavior of the different classes ensures high specificity and structural elucidation down to sub-species level.

In conclusion, we offer a comprehensive lipidomic analysis of three cortical sections of ob/ob mouse renal tubules, and we show that leptin deficiency profoundly and differentially affects the lipidomic composition of three tubular segments studied before the appearance of tubular lesions. This valuable resource provides unparalleled information that advances our understanding of tubular changes under the influence of pathological conditions associated with obesity, and will serve as a basis for generating pathophysiological hypotheses.

## Materials and methods

### Animal experiments

Experiments were performed on five 12 weeks male C57BL/6jRj wild-type and obese (B6.V-Lep ob/ob JRj) mice obtained from the Janvier laboratory and maintained at the Centre d’Explorations Fonctionnelles of the Cordeliers Research Center (agreement no. A75-06-12) until euthanasia for the isolation of kidney segments. Overdose of ketamine–xylazine was given intraperitoneally for anesthesia and euthanasia, followed by exsanguination by cardiac puncture and kidney harvest. We chose the minimum number of animals necessary to obtain robust and replicated data, while limiting the number of animals to be sacrificed as much as possible. The animals were either obese or lean, and did not receive any treatment, so there was no randomisation. No blinding was done, as the animals were obese or lean.

### Tubule segments isolation

Isolation of PCT, CTAL and CCD was performed according to localization in the kidney (cortex vs. medulla) and well-defined morphologic characteristics under binocular loupes after kidney treatment with Liberase (Sigma-Aldrich, St. Quentin Fallavier, France) [11]. Tubule length was measured using visilog software (Noesis, Courtabeuf, France), and pools of 150 mm (corresponding approximately to 150 segments) were transferred to 500 µL of PBS, rinsed 3 times, centrifuged (600 × *g*, 5 min), and resuspended in 150 mM ammonium bicarbonate. Samples were frozen in liquid nitrogen and stored at −80 °C until lipid extraction.

### Polymerase chain reaction

RNA was extracted from a small portion of the segments using the RNeasy micro kit (Qiagen, Hilden, Germany). mRNA was then reverse transcribed into cDNA (Roche Diagnostics, France) according to the manufacturer’s instructions and real-time PCR was performed on a LightCycler (Roche Diagnostics, France). No signal was detected in samples that did not undergo reverse transcription or in blank runs without cDNA. In each run, a standard curve was generated by serial dilution of the stock cDNA. To verify the quality of our isolation process, we measured the expression of specific markers for each isolated segment, namely *Cldn2* for PCT, *Slc12a1* (=*Nkcc2)* for CTAL and *Aqp2* for CCD using the following primers: *Rpl26* (NM_009080) F_GCTAATGGCACAACCGTC and R_TCTCGATCGTTTCTTCCTTGTAT; *Cldn2*NM_016675 (F_CAGTATGTCCAGACTGCATTG and R_AGATGGCCTGAGAAGGG), *Slc12a1* (NM_009194) F_GAGATTGGCGTGGTCATAGTCAGAA and R_TGCTGCTGATGTTGCCGTCTTT; *Aqp2* (NM_009699) F_GAGCGGGCTGGATTCATGGAG and R_CCTGTGACTGTGGCGTGCCTG). Primers for *Acsl4* were F_CTT CCT CTT AAG GCC GGG AC and R_TCT CTT TGC CAT AGC GTT TTT AGA. The expression of the housekeeping gene *Rpl26* was used to normalize the results.

The sequence of the primers used to monitor the ferroptosis-related genes are *Rpl13a*: 5’-CCCTATGACAAGAAAAAGCGGA-3’ and 5’-TTTCCTTCCGTTTCTCCTCCAG-3’; *Slc7A11*: 5’-ACTGTTCGGTCGTGACTTCC-3’ and R 5’-AATACGGAGCCTTCCACGAG-3’; *Gpx4:* 5’GCAACCAGTTTGGGAGGCAGGAG-3’ and 5’-CCTCCATGGGACCATAGCGCTTC-3’; *Fsp1*: 5’-GGCCTCTGCTTCATGGAGTT -3’ and 5’-CTGGCTAGCAATGGTGCTCT-3’; *Lpcat3:* 5’-CCCATGCACGTTGCCTTATC-3’ and 5’-AGAAAGCAGTCTGGGATGCTA-3’. The expression of the housekeeping gene *Rpl13a* was used to normalize the results.

### Lipidomics

For Lipidomics analysis, 150 mm of isolated tubules were spiked with 1.40 μL of internal standard lipid mixture containing 500 pmol of Chol-d6, 100 pmol of Chol-16:0-d7, 100 pmol of DG 17:0-17:0, 50 pmol of TG 17:0-17:0-17:0, 100 pmol of SM 18:1;2-12:0, 30 pmol of Cer 18:1;2-12:0, 30 pmol of GalCer 18:1;2-12:0, 50 pmol of LacCer 18:1;2-12:0, 300 pmol of PC 17:0-17:0, 50 pmol of PE 17:0-17:0, 50 pmol of PI 16:0-16:0, 50 pmol of PS 17:0-17:0, 30 pmol of PG 17:0-17:0, 30 pmol of PA 17:0-17:0, 40 pmol of Gb3 18:1;2-17:0, 25 pmol of GM3 18:1;2-18:0-d5, 25 pmol of GM2 18:1;2-18:0-d9, 25 pmol of GM1 18:1;2-18:0-d5, 30 pmol of LPA 17:0, 30 pmol of LPC 12:0, 30 pmol of LPE 17:1 and 30 pmol of LPS 17:1 and subjected to lipid extraction at 4 °C, as described elsewhere [41]. Briefly, the sample was dissolved in 200 μL of 155 mM ammonium bicarbonate and then extracted with 1 mL of chloroform-methanol (10:1) for 2 h. The lower organic phase was collected, and the aqueous phase was re-extracted with 1 mL of chloroform-methanol (2:1) for 1 h. The lower organic phase was collected and evaporated in a SpeedVac vacuum concentrator. Lipid extracts were dissolved in 100 μL of infusion mixture consisting of 7.5 mM ammonium acetate dissolved in propanol:chloroform:methanol [4:1:2 (vol/vol)]. Samples were analyzed by direct infusion in a QExactive mass spectrometer (Thermo Fisher Scientific) equipped witha TriVersa NanoMate ion source (Advion Biosciences). 5 µL of sample were infused with gas pressure and voltage set to 1.25 psi and 0.95 kV, respectively.

DG, TG and CE were detected in the 10:1 extract, by positive ion mode FTMS as ammonium aducts by scanning m/z = 580–1000 Da, at R_m/z = 200_ = 280 000 with lock mass activated at a common background (m/z = 680.48022) for 30 seconds. Every scan is the average of 2 micro-scans, automatic gain control (AGC) was set to 1E6 and maximum ion injection time (IT) was set to 50 ms. For fatty acid profiling of DG and TG, a parallel reaction monitoring was performed with an inclusion list of m/z = 580–1000 Da, at NCE of 20 and R_m/z = 200_ = 17500 for 30 s. Every scan is the average of 2 micro-scans, automatic gain control (AGC) was set to 1E6 and maximum ion injection time (IT) was set to 64 ms.

PC, PC O, OxPC, Cer, GlcCer, LPC and LPC O were detected as acetate adducts while PG, OxPG PE, OxPE, PE O, LPE and LPE O were detected as deprotonated adducts and CL was detected as doubly deprotonated adduct in the 10:1 extract, by negative ion mode FTMS, after polarity switch by scanning m/z = 420–1050 Da, at R_m/z = 200_ = 280,000 with lock mass activated at a common background (m/z = 529.46262) for 30 s. Every scan is the average of 2 micro-scans, automatic gain control (AGC) was set to 1E6 and maximum ion injection time (IT) was set to 50 ms. For FA profiling of PC, OxPC O, PC O, PE, OxPE, PE O, PG and OxPG a parallel reaction monitoring was performed with an inclusion list of m/z = 590–940 Da, at NCE of 35 and R_m/z = 200_ = 17,500 for 72 s. Every scan is the average of 2 micro-scans, automatic gain control (AGC) was set to 1E5 and maximum ion injection time (IT) was set to 64 ms.

LacCer and Gb3 were detected as protonated ions and Gb4 was detected as ammoniated adduct in the 2:1 extract in positive ion mode FTMS by scanning m/z = 800–1600 Da, at R_m/z=200_ = 280,000 with lock mass activated at a common background (m/z = 1,194.8179) for 30 s. GM1, GM2, andGM3 were detected as deprotonated ions in the 2:1 extract in negative ion mode after polarity switch in FTMS by scanning m/z = 1100–1650 Da, at R_m/z = 200_ = 280,000 with lock mass activated at a common background (m/z = 1175.7768) for 30 s. Every scan is the average of two micro-scans, AGC was set to 1E6 and IT was set to 50 ms in both polarities.

PA, PI, OxPI, PS, OxPS, LPA, and LPS were detected as deprotonated ions in the 2:1 extract in negative ion mode in FTMS by scanning m/z = 400–1100 Da, at R_m/z = 200_ = 280,000 with lock mass activated at a common background (m/z = 529.4626) for 30 s. Every scan is the average of two micro-scans, AGC was set to 1E6 and IT was set to 50 ms. For FA profiling of PA, PI, OxPI, PS and OxPS a parallel reaction monitoring was performed with an inclusion list of m/z = 590–940 Da, at NCE of 35 and R_m/z = 200_ = 17500 for 84 s. Every scan is the average of 2 micro-scans, automatic gain control (AGC) was set to 1E5 and maximum ion injection time (IT) was set to 64 ms.

All data was acquired in centroid mode. All lipidomics data were analyzed with the lipid identification software, LipidXplorer [42]. Lipid identification criteria and quantification strategy are thoroughly described elsewhere [43]. Tolerance for MS and MSMS identification was set to 2 and 5 ppm, respectively. Data post-processing and normalization to internal standards were done manually in Excel. The lipids contents are normalized to the total membrane lipid identified (excluding CE and TG) and expressed as a proportion of the whole lipid content in a sample. Data analysis was performed in the MetaboAnalyst 5.0 software [44]. Samples were normalized by sum and each metabolite level was adjusted by autoscaling.

### Statistical analysis

Graphs and statistical analyses were generated using GraphPad Prism 9 software (GraphPad Software, Inc.). Data are presented as mean ± standard error. Unpaired 2-sample *t*-tests were used to determine a significant difference between two groups and were 2-tailed. Multiple comparison within the same group were performed with *T* tests corrected with the Holm-Šídák method. Multiple comparison within different groups were performed with Oneway ANOVA corrected with the Šídák or Dunnett’s multiple comparison test. A *p*-value < 0.05 was considered a statistically significant difference.

### Ethics declarations

All experimental protocols and all methods were conducted according to French veterinary guidelines and those formulated by the European Commission for experimental animal use (L358–86/609EEC).

The experimental protocol was approved by the French Ministère de la Recherche (project no. 9499-201704031404679 v4).

The animals were kept at CEF (Centre d’Explorations Fonctionnelles) of the Cordeliers Research Center, Agreement no. A75-06-12). All methods are reported in accordance with ARRIVE guidelines

## Data Availability

The analytical process for lipidomics according to lipidomic standards (https://lipidomicstandards.org/reporting_checklist/) has been deposited and is publicly available as of the date of publication (10.5281/zenodo.10889906). Raw data of all lipids identified with their m/z values has been deposited and is publicly available as of the date of publication (10.5281/zenodo.14201781).
